# Virtual Evaluation of Hematoxylin & Eosin via Digital Pathology Survey (VEED) Project: Results from a Non-Inferiority Study of a Tabs-Based Staining Method

**DOI:** 10.3390/diagnostics16121868

**Published:** 2026-06-16

**Authors:** Lorenzo Nibid, Erica Iannaccone, Elisabetta Maffei, Veronica Vicomandi, Martina D’Angelo, Cristiana Bellan, Bruna Cerbelli, Giorgio Cazzaniga, Vincenzo L’imperio, Albino Eccher, Giuseppe Nicolò Fanelli, Alessandro Gambella, Luca Mastracci, Giuseppe Ingravallo, Stefano Marletta, Francesco Merolla, Pasquale Pisapia, Luisella Righi, Silvia Uccella, Mariavittoria Vescovo, Roberto Virgili, Alessandro Caputo, Giuseppe Perrone

**Affiliations:** 1 Research Unit of Anatomical Pathology, Department of Medicine and Surgery, Università Campus Bio-Medico Di Roma, 00128 Rome, Italy; erica.iannaccone@unicampus.it; 2 Anatomical Pathology Operative Research Unit, Fondazione Policlinico Universitario Campus Bio-Medico, 00128 Rome, Italy; veronica.vicomandi@unicampus.it (V.V.); martina.dangelo@policlinicocampus.it (M.D.); m.vescovo@policlinicocampus.it (M.V.); r.virgili@policlinicocampus.it (R.V.); 3 Pathology, Cytopathology, and Molecular Diagnostics Unit, University Hospital of Salerno, 84131 Salerno, Italy; maffeielisabetta@gmail.it (E.M.); alcaputo@unisa.it (A.C.); 4 Department of Medical Biotechnologies, Section of Pathological Anatomy, University of Siena, 53100 Siena, Italy; cristiana.bellan@unisi.it; 5 Department of Radiological, Oncological and Pathological Sciences, Sapienza University of Rome, 00161 Rome, Italy; bruna.cerbelli@uniroma1.it; 6 Department of Medicine and Surgery, Pathology, IRCCS Fondazione San Gerardo dei Tintori, University of Milano-Bicocca, 20126 Milan, Italy; giorgio9cazzaniga@gmail.com (G.C.); vincenzo.limperio@unimib.it (V.L.); 7 Department of Medical and Surgical Sciences for Children and Adults, University of Modena and Reggio Emilia, University Hospital of Modena, 41124 Modena, Italy; albino.eccher@unimore.it; 8 Division of Pathology, Department of Translational Research and New Technologies in Medicine and Surgery, University of Pisa, 56126 Pisa, Italy; nicolo.fanelli@unipi.it; 9 First Division of Pathology, Department of Oncology, Pisa University Hospital, 56126 Pisa, Italy; 10 Department of Pathology and Laboratory Medicine, Weill Cornell Medical College, New York, NY 10065, USA; 11 Pathology Unit, Department of Surgical Sciences and Integrated Diagnostics (DISC), University of Genoa, 16126 Genoa, Italy; alessandro.gambella@unige.it (A.G.); luca.mastracci@unige.it (L.M.); 12 IRCCS Azienda Ospedaliera Metropolitana (IRCCS AOM), Plesso IRCCS San Martino, 16126 Genoa, Italy; 13 Section of Molecular Pathology, Department of Precision and Regenerative Medicine and Ionian Area (DiMePRe-J), University of Bari “Aldo Moro”, 70121 Bari, Italy; giuseppe.ingravallo@uniba.it; 14 Department of Biomedical Science, Humanitas University, 20072 Milan, Italy; stefano.marletta@humanitascatania.it (S.M.); silvia.uccella@hunimed.eu (S.U.); 15 Division of Pathology, Humanitas Istituto Clinico Catanese, 95045 Catania, Italy; 16 Department of Medicine and Health Sciences “V. Tiberio”, University of Molise, 86090 Campobasso, Italy; francesco.merolla@unimol.it; 17 Department of Public Health, University of Naples Federico II, 80138 Naples, Italy; pasquale.pisapia@unina.it; 18 Pathology Unit, Department of Oncology at San Luigi Hospital, University of Torino, 10124 Torino, Italy; luisella.righi@unito.it; 19 Department of Pathology, IRCCS Humanitas Research Hospital, 20089 Milan, Italy

**Keywords:** digital pathology, pathology, histology, tissue stain, H&E, hematoxylin, eosin, pre-analytics

## Abstract

**Background/Objectives:** Despite hematoxylin and eosin (H&E) staining remaining the cornerstone of histopathological diagnosis, substantial intra- and inter-laboratory variability persists. This issue is increasingly relevant in Digital Pathology, where staining inconsistency may affect whole-slide image interpretation and the performance of image analysis algorithms. In the present work, we evaluated the diagnostic adequacy and non-inferiority of a novel tabs-based H&E histochemical staining method compared with conventional liquid reagents. **Methods:** Fifty formalin-fixed paraffin-embedded tissue samples from routine practice were sectioned in duplicate and stained either conventionally or using H&E Stain Tabs. After slide review, 14 representative tissue samples were selected, scanned at 40× magnification, and used to generate 24 matched image pairs at different magnifications. A blind online survey was completed by 13 expert pathologists using high-quality monitors. Participants assessed overall staining preference and rated stromal, epithelial, cytoplasmic, and nuclear staining quality. Non-inferiority was tested using a predefined margin of −0.10, and paired rating differences were analyzed using the Wilcoxon signed-rank test. **Results:** Across 312 paired evaluations, the tabs-based method was preferred in 120 cases (38.5%), conventional staining in 118 cases (37.8%), and no preference was expressed in 74 cases (23.7%). The tabs-based method met the criterion for non-inferiority compared with standard staining (z = 2.7). Rating-scale analysis showed significantly better stromal evaluation with the tablet-based method (z = 2.638; *p* = 0.008), whereas no significant differences were observed for epithelial, cytoplasmic, or nuclear staining. All evaluated images were considered diagnostically adequate. **Conclusions:** The tabs-based H&E stain was non-inferior to the conventional method and showed particularly favorable performance in the assessment of stromal components. These findings support its potential role in improving staining reproducibility and standardization, particularly in Digital Pathology workflows where pre-analytical and analytical consistency is critical.

## 1. Introduction

Hematoxylin and eosin (H&E) staining has been the cornerstone of anatomic pathology for more than a century and remains the most adopted tissue-staining method worldwide [1]. Adequate staining highlights cellular and stromal components, thereby supporting histological examination, improving diagnostic confidence and reproducibility, and reducing the need for additional sectioning or immunohistochemistry in case of morphological uncertainty [2].

Nevertheless, a certain intra- and inter-laboratory variability in H&E staining is frequently observed and may be attributable to several causes. First, both hematoxylin and eosin are commonly supplied as pre-diluted solutions, which may be subject to degradation and influenced by external factors such as temperature and storage time. These issues are also associated with increased shipping and storage costs, as well as a potentially relevant environmental impact. In addition, tissue-section thickness can affect staining quality. Finally, staining variability may also reflect individual pathologists’ preferences regarding the balance between hematoxylin and eosin [3,4].

Despite valuable efforts by organizations such as the College of American Pathologists (CAP) and the United Kingdom National External Quality Assessment Service (UK NEQAS) to improve staining quality, universally accepted objective standards for assessing H&E staining adequacy are still lacking [2,4,5,6]. In routine practice, pathologists therefore assess H&E-stained sections largely based on experience and subjective preference. This issue has become increasingly relevant with the adoption of whole-slide imaging (WSI), as staining variability may affect both digital slide interpretation and the performance of artificial intelligence algorithms. Several studies have investigated digital normalization or virtual H&E staining of WSIs [7,8,9,10,11,12,13,14]. However, to the best of our knowledge, no studies have specifically addressed standardization at the staining-procedure level.

In this study, we evaluated a novel tabs-based H&E histochemical staining method designed to improve staining reproducibility. The evaluation was performed virtually by a selected group of expert pathologists using a Digital Pathology-based survey.

## 2. Materials and Methods

### 2.1. Sample Cohort

**Figure 1:**
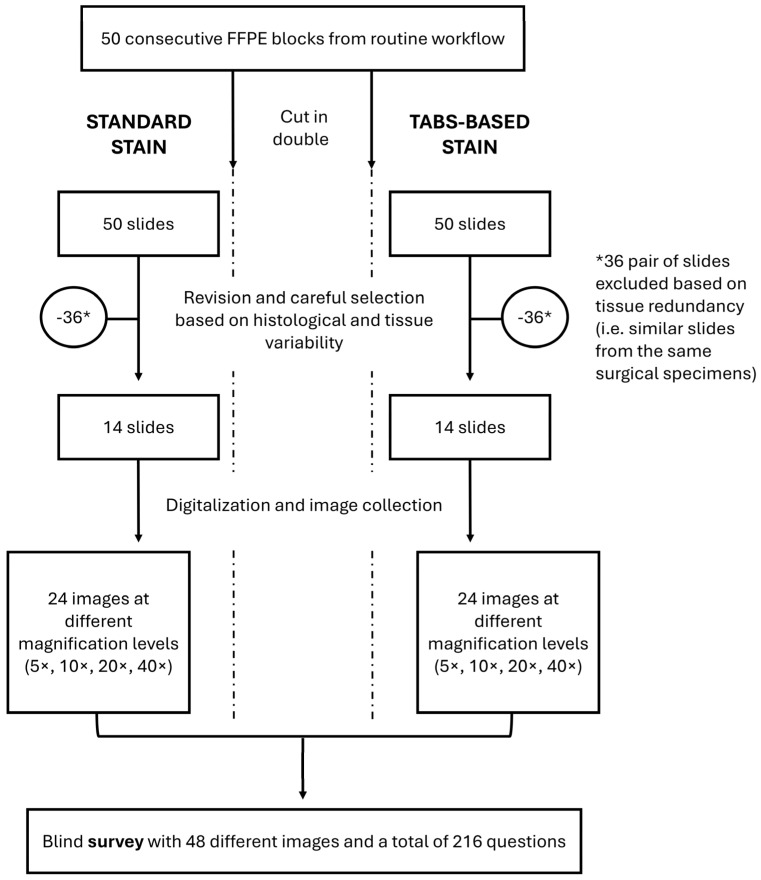
Cohort selection and tissue-sample workflow. A total of 50 FFPE tissue slides were sectioned in duplicate: 50 stained conventionally and 50 by the Stain Tabs. All 100 slides obtained were revised and, based on tissue sample variability, 14 pairs of slides were selected to obtain 24 image pairs at different magnification levels. Therefore, 48 images were used to conduct a blind survey.

A total of 50 consecutive formalin-fixed and paraffin-embedded (FFPE) tissue samples from routine clinical practice were sectioned in duplicate at 5 µm thickness. For each sample, one section was stained conventionally, whereas the paired section was stained using Stain Tabs (Diapath S.p.A., Martinengo BG, Italy) for a total of 100 slides. After a careful review of tissue slides by three pathologists (L.N., E.I., and E.M.), 14 samples were selected based on histological and tissue variability, and 28 slides were digitized at 40× magnification by NanoZoomer 360SD (Hamamatsu Photonics, Shizuoka, Japan). A total of 48 images at different magnification levels (5×, 10×, 20× and 40×) were obtained focusing on both normal and pathological tissue features. Figure 1 represents the tissue-sample workflow.

All tissue samples and magnification levels used are reported in Table 1.

### 2.2. Staining Methods

FFPE tissue samples were sectioned at 3–5 μm thickness, mounted on glass slides, and dried prior to staining. H&E staining was performed using an automated staining system (Giotto, Diapath S.p.A., Martinengo BG, Italy), following two protocols for standard and Stain Tabs-based tissue stain, respectively. All slides were first deparaffinized in three consecutive xylene baths (10 min each) and rehydrated through graded ethanol solutions (99% for 1 min, followed by two steps in 95% ethanol for 1 min each), then rinsed in distilled water for 2 min. In the standard protocol, hematoxylin (CP813, GILLII, Diapath S.p.A., Martinengo BG, Italy) staining was carried out for 3 min, followed by washing in running tap water for 5 min, in ammonia water for 40 s, in running tap water for 2 min and in 95% ethanol for 1 min. Eosin (CO355, Eosin G OR Y alcoholic 0.5%, Diapath S.p.A., Martinengo BG, Italy) counterstaining was carried out for 3 min. In case of Stain Tabs, hematoxylin and eosin solutions were obtained by overnight dilution of 1 hematoxylin and 1 eosin Stain Tab in 800 mL of distilled water (400 mL each for hematoxylin and eosin) and added to the Giotto stainer after proper filtration. Slides were left in hematoxylin for 5 min, followed by washing in running tap water for 5 min. Slides were then treated with ammonia water for 1 min for bluing, rinsed again in running tap water for 2 min, and in distilled water for 1 min. Then, eosin counterstaining was carried out for 3 min. In both protocols, slides were subsequently dehydrated through graded alcohols and cleared in xylene and coverslipped using a permanent mounting medium.

### 2.3. Tissue Stain Evaluation

The new staining method was compared to the standard staining technique by a non-inferiority approach. We identified new simple parameters inspired by the CAP H&E Troubleshooting Guide and UK NEQAS staining evaluation criteria. Therefore, an online survey was developed using the JotForm platform (Jotform Inc., San Francisco, CA, USA) to allow tissue stain evaluation. The survey was nominal and all participants were asked to provide their surname, given name, email address, and the monitor used for the evaluation. Participants had to express preference for pairs of images stained conventionally and by tabs, even specifying if they had no preferences; this was done in order to collect data on the overall stain preference (in this evaluation the overall intensity, specificity, and uniformity of H&E were evaluated, along with the background, presence of particles, and contrast). Moreover, readers were asked to give a rating scale (from 1 to 3) for the epithelial/inflammatory and stromal components to evaluate the interactions of hematoxylin and eosin in these compartments. Similarly, a rating scale (from 1 to 3) was adopted for the nuclear and cytoplasmic staining in each image, representing an indirect parameter of adequacy for hematoxylin and eosin, respectively. Therefore, the survey comprised a total of 216 questions. A pilot test of the survey was first conducted during the 37th European Congress of Pathology (ECP) by eight volunteer pathologists under suboptimal environmental conditions to verify its usability and technical performance.

The survey was conducted by 13 expert pathologists from different centers (C.B., G.C., B.C., A.E., G.N.F., A.G., G.I., S.M., F.M., P.P., L.R., S.U., M.V.) using high-quality monitors (screen size ranging from 27 to 75 inches with a minimum resolution of 3840 × 2160—8 MP). The screens used are summarized in Table 2. The survey is available at https://form.jotform.com/251474857931366 (accessed on 14 June 2026).

### 2.4. Statistical Analysis

A non-inferiority test was performed to compare the proportion of subjects selecting the new option versus the standard option. Responses were categorized as New, Standard, or None, with all categories included in the denominator for the calculation of proportions. The proportions of New (pN) and Standard (pS) choices were computed over the total number of observations, and the difference in proportions (d = pN − pS) was estimated. The variance and standard error of the difference were calculated assuming a binomial distribution. Non-inferiority was assessed using a one-sided z-test with a predefined non-inferiority margin of −0.10. The null hypothesis of inferiority was rejected when the z-statistic exceeded the critical value of 1.645, corresponding to a one-sided significance level of 0.05. Descriptive statistics were used to summarize response frequencies. The Wilcoxon signed-rank test was adopted to assess differences between the two groups in terms of stroma, epithelium, cytoplasm and nuclear rating scale; number of positive and negative differences were compared in case of significance to assess which method performed better. Statistical difference was defined as *p* < 0.05 and analyses were performed using IBM SPSS Statistics 27 (IBM Corp., Armonk, NY, USA).

## 3. Results

**Figure 2:**
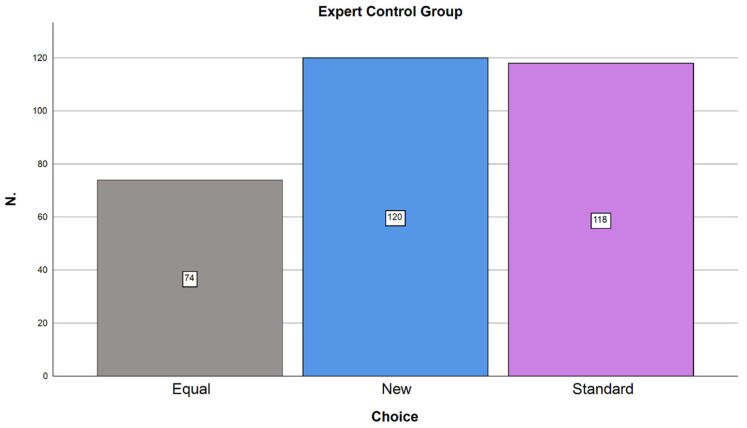
Histogram representing preference evaluation among standard (violet) and new tabs-based tissue staining (blue). The gray column represents the number of cases in which no preference between the two methods was registered.

In the preference analysis, the expert cohort of 13 pathologists evaluated 24 pairs of images, yielding a total of 312 paired assessments. No missing answers were recorded, and all evaluations were performed using high-quality monitors. Overall, the tablet-based staining method was preferred in 120/312 evaluations (38.5%), conventional staining in 118/312 evaluations (37.8%), and no preference was expressed in 74/312 evaluations (23.7%). Statistical analysis demonstrated the non-inferiority of the tablet-based method (z = 2.7). Preference results are summarized in Figure 2. By contrast, preliminary data from the eight volunteer pathologists who completed the pilot test under suboptimal environmental conditions did not demonstrate non-inferiority (standard preference: 83/192, 43%; tabs preference: 54/192, 28.1%; no preference: 27/192, 14.1%; missing data: 28/192, 14.6%; z = 1.43).

**Figure 3:**
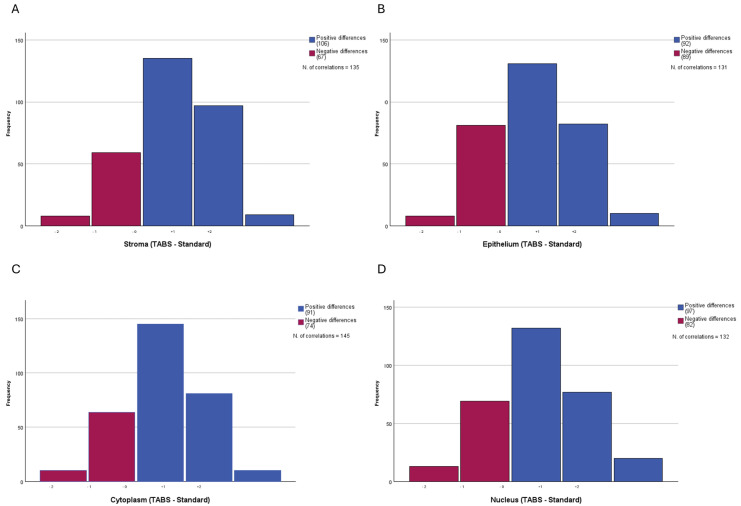
Comparison of stroma (**A**), epithelium (**B**), cytoplasm (**C**) and nuclear (**D**) rating scale among tabs-based and standard staining methods. Red columns represent negative differences while blue columns represent positive differences between tabs-based and standard stains. The Wilcoxon signed-rank test revealed a statistically significant difference between the two methods in terms of stromal (**A**) rating (z = 2.638; *p* = 0.008), highlighting a predominance of positive ranks (106) over negatives (67).

Considering the rating scale, Stain Tabs outperformed the standard staining method in terms of stromal evaluation (z = 2.638; *p* = 0.008) while no statistically significant differences were found for epithelium (z = 0.334; *p* = 0.738), cytoplasm (z = 1.107; *p* = 0.268) and nuclei (z = 1.340; *p* = 0.180). In detail, 106 positive and 67 negative differences were found for stromal rating scale, 92 positive and 89 negative differences for the epithelium, 91 positive and 74 negative differences for the cytoplasm and 97 positive and 82 negative differences for the nuclei. Stromal score 1 (poor quality) was observed in 13/312 (4.2%) tabs-based stained slides and 16/312 (5.1%) standard-stained slides. Score 2 (intermediate quality) was assigned to 89/312 (28.5%) tabs-based images and 126/312 (40.4%) standard-stained slides. Score 3 (high quality) was recorded more frequently in tabs-based slides (206/312, 66.0%) compared with standard-stained slides (170/312, 54.5%). Stromal data were missing in 4/312 images (1.3%). For the epithelial rating scale, score 1 was reported in 14/312 (4.5%) tabs-based stained slides versus 20/312 (6.4%) standard-stained slides. Score 2 was assigned in 102/312 (32.7%) tabs-based images and 95/312 (30.5%) standard-stained slides. High-quality epithelial staining (score 3) was observed in 196/312 (62.8%) tabs-based and 197/312 (63.1%) standard-stained slides. Regarding cytoplasmic assessment, score 1 was registered in 17/312 (5.4%) tabs-based stained slides and 22/312 (7.1%) standard-stained slides. Score 2 was observed in 103/312 (33.0%) tabs-based images and 110/312 (35.3%) standard-stained slides. Score 3 was more frequent in tabs-based slides (191/312, 61.2%) than in standard-stained slides (179/312, 57.4%). Cytoplasmic data was missing in 2/312 images (0.6%). For nuclear staining, score 1 was recorded in 22/312 (7.1%) tabs-based stained slides compared with 33/312 (10.6%) standard-stained slides. Score 2 was assigned in 103/312 (33.0%) tabs-based images and 102/312 (32.7%) standard-stained slides. High-quality nuclear staining (score 3) was observed in 187/312 (59.9%) tabs-based slides and 176/312 (56.4%) standard-stained slides. Nuclear missing data were identified in 1/312 images (0.3%). Results are summarized in Table 3 and Figure 3.

## 4. Discussion

H&E staining has been used in histopathology for more than a century and remains essential for the morphological evaluation of normal and pathological tissues. However, staining quality can affect diagnosis, and it mainly relies on hematoxylin and eosin balance. In particular, hematoxylin intensity must be strong enough to highlight nuclear details already at a medium power, while contemporarily avoiding chromatin granularity loss or excessive cytoplasmic and connective tissue staining. Similarly, eosin must be selective enough to demonstrate tissue morphology and various cellular components: not too weak to affect the low power evaluation and not too strong to obscure the color and details of nuclear stain. Despite that, staining techniques suffer from high intra- and inter-laboratory variability, and standardized open-access or widely shared criteria for stain evaluation are still not available. Ultimately, these features and considerations represent critical limitations for standardization and improvement of Digital Pathology.

Nowadays, an increasing number of anatomic pathology departments are adopting Digital Pathology workflows to increase the traceability and control of laboratory phases. This implies the use of traceability systems and slide scanners to obtain WSIs, thus enabling observation by viewer software on a computer instead of traditional optical light microscopes [15,16,17,18]. In this context, standardization of histopathological techniques (including staining) is pivotal to minimize technical variability and maximize the benefits of digital transformation. In detail, the adoption of WSI opened the door for the application of deep learning and machine learning approaches aimed at extracting diagnostic and predictive information from histopathological images [19,20,21]. Interestingly, a quantitative analysis revealed that about 40% of tissue samples from 247 laboratories undertaken by the UK NEQAS CPT and National Pathology Imaging Co-operative (NPIC) registered clearly perceptible staining differences; notably, this percentage could be even higher in laboratories that do not undergo voluntary H&E quality control. Considering these data and the possibility that image analysis performance may be affected by staining variability, several studies have investigated the potential for digital standardization of H&E-stained tissue slides; based on this, standardization tools are commercially available [7,8]. Similarly, other authors developed a deep learning-based virtual staining method utilized directly on unstained slides or fresh (not formalin-fixed) tissues. The limitations of these approaches mainly lie in the reliance on ground-truth identification and scanner adoption; in fact, most of these models are trained and tested using slides, staining reagents, and digital scanners from a limited number of centers, and are therefore unable to capture real-world variability. Thus, real-life feasibility and reproducibility of these techniques should still be demonstrated [11,12,13,22,23,24].

**Figure 4:**
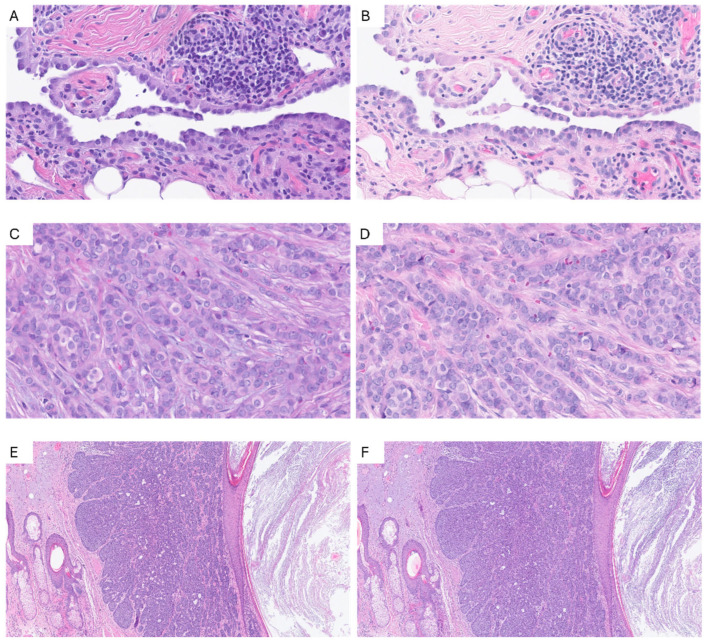
H&E images from pleura (**A**,**B**; 40× magnification), lobular carcinoma of the breast (**C**,**D**; detail from 20× magnification) and basal cell carcinoma (**E**,**F**; 5× magnification). Slides in (**A**,**C**,**E**) were stained conventionally whereas images in (**B**,**D**,**F**) were stained by tabs. Note the nuclei and nucleoli definition in (**B**,**D**) and the higher contrast between epithelium and stroma in (**F**).

In our study, we developed a survey for the histopathological evaluation of an innovative tabs-based H&E staining method. The new product consists of one eosin and one hematoxylin tab which must be diluted in distilled water. In contrast with digital staining, the new method represents a chemical stain based on affinity between acids and bases, and is therefore not affected by ground-truth-related limitations inherent to virtual staining approaches; this staining technique was developed to limit the risk of decay that affects all pre-diluted solutions, and to increase intra- and inter-laboratory reproducibility. Since there are no validated and open-access criteria for the adequacy assessment of H&E tissue stains, we evaluated the non-inferiority of the new staining method via a user-friendly on-line form that has been distributed to a selected group of expert pathologists. The form was designed to capture the synthesis of several described parameters in few questions. Our findings demonstrate that tabs-based staining provides high-quality histological images while maintaining morphological detail comparable to that achieved with conventional pre-diluted H&E staining methods. Importantly, in all cases the staining was adequate for a histopathological diagnosis and statistical analysis demonstrated the non-inferiority of the new staining technique (z = 2.7). In detail, no preference (74/312; 23.7%) and Stain Tabs preference (120/312; 38.5%) were registered in 194/312 answers (62.2%), while standard stain preference was observed in 118/312 (37.8%) cases. Moreover, the new staining method outperformed conventional staining in terms of stromal rating scale (z = 2.638; *p* = 0.008). This result is probably due to the paler hematoxylin stain which enhances the stromal component, while not compromising nuclear evaluation and making nucleoli and mitoses more easily visible. In fact, the tabs-based method obtained a higher number of nuclei 2 and 3 scores and a lower number of 1 scores compared with the standard method, globally ensuring excellent- and good-quality stains in most cases and a low number of poor-quality stains (Figure 4).

The tabs-based histochemical approach appears to be compatible with routine Digital Pathology workflows, allowing straightforward implementation without the need for substantial changes to standard laboratory procedures. It only requires the preparation of tab dilution and hematoxylin filtration as additional steps, offering substantial advantages in terms of storage and shipping, as well as potential benefits in standardization. In addition, our data highlight the potential impact of dedicated equipment (in particular, monitor quality) and environmental context on WSI evaluation. In fact, it was possible to demonstrate non-inferiority using only the expert cohort that completed the survey in an optimal environmental setting, using high-quality monitors, in contrast to the initial pilot analysis in suboptimal conditions. These findings emphasize the importance of monitor quality as an analytical variable in Digital Pathology [25,26].

The limitations of our study lie primarily in the incomplete representation of tissues; for example, the survey did not include tissues from the lung, bladder, lymph nodes, bone, and central nervous system. Additionally, all specimens were collected and stained at a single institution, precluding assessment of inter-laboratory variability. Future studies will include a multicenter evaluation involving independent laboratories, different staining platforms, different tissue samples and diverse workflow settings to further validate the present findings and investigate both intra- and inter-laboratory variability. To this end, quantitative image analysis may be incorporated in subsequent investigations. Nevertheless, these findings provide a pivotal foundation for future investigations using the same described approach, with broader inclusion of tissue types and samples from multiple centers.

Nevertheless, to the best of our knowledge, this is the first attempt to develop a Digital Pathology-based platform for H&E quality assessment. Our survey proposed a compromise between a detailed and user-friendly evaluation, avoiding a huge number of specific questions for each single image, and considering the judgment of a selected group of expert pathologists. The value of our results is reinforced by the strict selection of the users: all highly experienced and respected Italian pathologists.

## 5. Conclusions

Given the lack of widely accepted standards for assessing H&E staining adequacy, we developed a user-friendly digital survey to evaluate the non-inferiority of a tablet-based H&E staining method. Our results demonstrated the non-inferiority of this method compared with conventional staining and showed particularly favorable performance in the assessment of stromal components.

In view of the increasing need for standardization in anatomic pathology departments, the development and dissemination of shared criteria for evaluating H&E-stained tissue sections should be strongly encouraged. H&E staining quality, monitor characteristics, and environmental conditions should be regarded as relevant pre-analytical and analytical variables in Digital Pathology. Careful control of these parameters may reduce intra- and inter-laboratory variability and further improve the effectiveness of Digital Pathology workflows and image analysis algorithms in routine clinical practice. Once non-inferiority has been established, further studies are needed to evaluate the impact of the tablet-based H&E staining method on staining variability, including both intra- and inter-laboratory reproducibility, supported by quantitative analytical assessments.

## Figures and Tables

**Table 1:** Tissues and magnification levels used in the survey.

N. Picture	Tissue	Pathology	Magnification
1	Skin	Actinic keratosis	10×
2	Skin	Basal cell carcinoma	5×
			20×
			40×
3	Skin	Dyskeratoma	20×
			40×
4	Prostate	Normal and prostate cancer	5×
			10×
			20×
			20×
			40×
5	Uterus	Leiomyoma	10×
6	Uterus	Adenomyosis	20×
7	Fallopian tubes	Normal	20×
8	Breast	Lobular carcinoma	20×
9	Breast	Apocrine metaplasia	10×
10	Kidney	Normal and chromophobe renal cell carcinoma	20×
			10×
11	Pleura	Pleural effusion	20×
			40×
12	Gallbladder	Cholecystitis	10×
			50×
13	Colon	Tubular adenoma	40×
14	Colon	Mesenteric panniculitis	20×

**Table 2:** High-quality screens used by the cohort of expert Italian pathologists.

Monitor	Inches	Resolution (Pixel)	N. Users
LG 32HL512D (LG Electronics, Seoul, Republic of Korea)	32	3840 × 2160	7
DELL U3223QE (Dell Technologies, Round Rock, TX, USA)	32	3840 × 2160	3
Barco MDPC-8127 (Barco, Kortrijk, Belgium)	27	3840 × 2160	1
Apple Studio Display (Apple Inc., Cupertino, CA, USA)	27	5120 × 2880	1
Samsung QLED 4K Ultra HD Q7F (Samsung Electronics, Suwon, Republic of Korea)	75	3840 × 2160	1

**Table 3:** Distribution of histological quality scores by tissue compartment.

	Stroma		Epithelium		Cytoplasm		Nuclei	
	TABS *n* (%)	Standard *n* (%)	TABS *n* (%)	Standard *n* (%)	TABS *n* (%)	Standard *n* (%)	TABS *n* (%)	Standard *n* (%)
**1**	13 (4.2)	16 (5.1)	14 (4.5)	20 (6.4)	17 (5.4)	22 (7.1)	22 (7.1)	33 (10.6)
**2**	89 (28.5)	126 (40.4)	102 (32.7)	95 (30.5)	103 (33.0)	110 (35.3)	103 (33.0)	102 (32.7)
**3**	206 (66.0)	170 (54.5)	196 (62.8)	197 (63.1)	191 (61.2)	179 (57.4)	187 (59.9)	176 (56.4)
**Missing**	4 (1.3)	0 (0.0)	0 (0.0)	0 (0.0)	1 (0.3)	1 (0.3)	0 (0.0)	1 (0.3)

## Data Availability

The aggregated data supporting the findings of this study are available from the corresponding author upon reasonable request. Individual reader-level data are not publicly available in order to protect participant confidentiality.
